# Leaf extract of *Garcinia atroviridis* promotes anti-heat stress and antioxidant effects in *Caenorhabditis elegans*

**DOI:** 10.3389/fphar.2024.1331627

**Published:** 2024-03-07

**Authors:** Sirithip Chuaijit, Chuchard Punsawad, Veronica Winoto, Waluga Plaingam, Itti Kongkaew, Atidtaya Phetcharat, Takafumi Ichikawa, Makoto Kubo, Fumitaka Kawakami, Aman Tedasen, Moragot Chatatikun

**Affiliations:** ^1^ Department of Medical Science, School of Medicine, Walailak University, Nakhon Si Thammarat, Thailand; ^2^ Research Center in Tropical Pathobiology, Walailak University, Nakhon Si Thammarat, Thailand; ^3^ Center of Excellence Research for Melioidosis and Microorganisms (CERMM), Walailak University, Nakhon Si Thammarat, Thailand; ^4^ Department of Chemical Engineering, Thammasat School of Engineering, Thammasat University Rangsit Campus, Rangsit, Pathum Thani, Thailand; ^5^ College of Oriental Medicine, Rangsit University, Rangsit, Pathum Thani, Thailand; ^6^ Department of Medical Technology, School of Allied Health Sciences, Walailak University, Nakhon Si Thammarat, Thailand; ^7^ Department of Regulation Biochemistry, Kitasato University Graduate School of Medical Sciences, Sagamihara, Japan; ^8^ Regenerative Medicine and Cell Design Research Facility, School of Allied Health Sciences, Kitasato, Sagamihara, Japan

**Keywords:** *Garcinia atroviridis*, oxidative stress, antioxidants, longevity, *Caenorhabditis elegans*

## Abstract

**Introduction:** *Garcinia atroviridis* has been used for traditional medicines, healthy foods and tea. The chemical compositions and biological activities of fruit, stem bark and root have been widely studied. However, the phytochemical components and the biological activities in *Garcinia atroviridis* leaves (GAL) are limited. This research aims to study the phytochemical components and the stress resistance effects of GAL in *Caenorhabditis elegans* (*C. elegans*).

**Methods:** To investigate the chemical components and antioxidant activities of GAL extract, the ethanol extract was characterized by liquid chromatography-quadrupole time-of-flight mass spectrometry (LC-QTOF MS) analysis and *C. elegans* was used to evaluate the effects of GAL extracts on longevity and stress resistance.

**Results and discussion:** The results revealed that the ethanol extract of GAL possesses free radical scavenging activities. Furthermore, GAL extract increased the lifespan of *C. elegans* by 6.02%, 15.26%, and 12.75% at concentrations of 25, 50, and 100 μg/mL, respectively. GAL extract exhibited improved stress resistance under conditions of heat and hydrogen peroxide-induced stress. The survival rates of GAL extract-treated worms were significantly higher than those of untreated worms, and GAL extract reduced reactive oxygen species (ROS) accumulation. Additionally, GAL extract treatment upregulated the expression of stress resistance-associated genes, including gst-4, sod-3, skn-1, and hsp16.2. GAL extract supplementation alleviated stress and enhanced longevity by inducing stress-related genes in *C. elegans*. The observed effects of GAL extracts may be attributed to the stimulation of oxidant enzymes mediated through DAF-16/FOXO and SKN-1/NRF2, as well as the enhancement of thermal defense in *C. elegans*. Collectively, this study provides the first evidence of the antioxidant activities of GAL and elucidates the underlying mechanisms of stress resistance.

## 1 Introduction

Mitochondria are the main producers of reactive oxygen species (ROS) during oxidative stress, a state characterized by an imbalance between the generation of ROS and the cellular antioxidant defenses. Excessive ROS levels are typically stemmed from mitochondrial activities related to oxygen metabolism. Moreover, ROS are fundamental elements in cellular activities such as gene transcription and immune response, with common types including hydroxyl radicals, superoxide, and hydrogen peroxide. However, an excess of ROS, due to environmental factors or nutritional deficiencies, can cause oxidative harm to biomolecules which in turn contributes to aging and degenerative diseases. Degenerative diseases pose a significant challenge to human health due to their intricate causes. ROS can damage proteins, lipids, and DNA, leading to various diseases such as Alzheimer’s and Parkinson’s. In addition, lipid peroxidation initiated by ROS can compromise cell membrane integrity, leading to cardiovascular disease, neurodegenerative disorders, and cancer. ROS-induced DNA damage can result in mutations and genomic instability, potentially leading to cancer. Additionally, mitochondrial dysfunction caused by ROS can further increase ROS production and lead to cell death, contributing to various degenerative diseases. ROS also activate inflammatory pathways, which are involved in degenerative conditions including arthritis and atherosclerosis. Targeting oxidative stress and its damage may be a promising therapeutic approach for degenerative diseases (Singh et al., 2019). Modulating the balance between ROS production and antioxidant defenses could help prevent and treat these diseases. Under normal physiological conditions, cellular integrity is protected by an antioxidant defense system that is chiefly made up of enzymatic components such as superoxide dismutase (SOD), catalase (CAT), and glutathione peroxidase (GPx). Collectively, these enzymes function to mitigate cellular damage induced by ROS (Ighodaro and Akinloye, 2018). Furthermore, antioxidants derived from natural products can scavenge excessive radicals, potentially extending lifespan and delaying the aging process. Numerous studies have reported the antioxidant properties of phytochemicals present in fruits and vegetables. For instance, mulberry leaf polyphenols have been found to reduce aging and regulate fat metabolism in specific organisms (Zheng et al., 2014). *Cassia fistula (L.)* exhibited significant antioxidant and neuroprotective properties *in vivo* (Thabit et al., 2018).

*Garcinia atroviridis* Griff. ex T. Anderson (*Garcinia atroviridis*) belongs to the tropical family Clusiaceae and is typically found in Thailand, Myanmar, and Malaysia. It is commonly known as “Asam Gelugor” or “Som-Khaek”. In Thailand, *G. atroviridis* products, such as capsules, fruit slices, and tea, are considered healthy food options (Shahid et al., 2022). The leaves of *G. atroviridis* have been historically employed in addressing a spectrum of ailments, encompassing stomach discomforts associated with pregnancy, earaches, dandruff, coughs, and constipation (Grosvenor et al., 1995). A preceding investigation delineated the composition of phytochemicals within the ethanolic extract of *G. atroviridis* leaves, revealing prominent constituents such as hexanoic acid, 9,12,15-octadecatrienoic acid, and octadecanoic acid. Notably, this extract exhibited antioxidative properties, including DPPH scavenging, OH scavenging, and reducing power activities, along with the capacity to inhibit the growth of *Staphylococcus aureus*, *Bacillus cereus*, *Escherichia coli*, and *Salmonella typhi*. Furthermore, administration of the ethanolic extract at a dosage of 200 mg/kg induced a reduction in body weight and decreased the levels of total cholesterol, triglyceride, and low-density lipoprotein cholesterol in hyperlipidemic rats fed a high-fat diet (Wairata et al., 2022). The aqueous extract of *G. atroviridis* leaves exhibited a notable phenolic content of approximately 0.21 ± 0.21 mgGAE/mg, alongside elevated levels of proteins, carbohydrates, and ash content. Additionally, this extract demonstrated antioxidative prowess through DPPH radical scavenging and ferric reducing antioxidant power activities (Nursakinah et al., 2012). Consumption of *G. atroviridis* leaf tea elicited a significant reduction in fasting blood glucose and triglyceride levels among obese adults over a span of 30 days (Lumbantobing et al., 2017a). Moreover, the methanolic extract of *G. atroviridis* leaves displayed broad-spectrum antibacterial activity against both Gram-positive (*Bacillus subtilis* B28&B29, methicillin-resistant *S. aureus*, *S. aureus*) and Gram-negative bacteria (*E. coli* and *Pseudomonas aeruginosa*), alongside antifungal efficacy against *Cladosporium herbarum*. This methanolic extract also showcased potent antioxidant activity via ferric thiocyanate and thiobarbituric acid methods, in addition to demonstratingantitumorr effects in Epstein-Barr virus-induced Raji cells (Mackeen et al., 2000). Notably, the methanolic extract exhibited significant inhibition of *Clostridium perfringens*-neuraminidase (NA), with an IC50 value of 36.17 g/mL, whereas the ethyl acetate extract demonstrated inhibitory activity against both NA (38.39 g/mL). Chemical analysis revealed the presence of naringenin and garcinia acid in the methanolic extract (Muchtaridi et al., 2022). However, there remains a paucity of evidence regarding the impact of the ethanolic extract of *G. atroviridis* leaves on longevity and resistance to heat stress.

*Caenorhabditis elegans* (*C. elegans*), a soil nematode, serves as a model organism for pharmacological studies, aging, and longevity (Jabeen et al., 2020). It has a short life cycle, a short lifespan and produces a large number of offspring. Furthermore, the genome of *C. elegans* has been fully sequenced, and a significant portion (60%–80%) of its genes have been identified as highly conserved homologs of human genes (Lai et al., 2000). In *C. elegans*, two crucial transcription factors, SKN-1/NRF2 and DAF-16/FOXO, play a vital role in the response to oxidative stress. The expression of SOD-3 and GST-4, two antioxidant enzymes encoded by SKN-1/NRF2 and DAF-16/FOXO, has been examined. SKN-1, analogous to the human NRF protein, is essential for regulating phase-II detoxification enzymes and antioxidant proteins such as SOD, GST, glutathione peroxidase (GPO), and NAD(P)H: quinone oxidoreductase (NQO-1). Aging and oxidative stress pathways in *C. elegans* have been the focus of numerous studies. For instance, Hyung An et al. discovered that SKN-1 mutants are sensitive to oxidative stress and exhibit shortened lifespans in *C. elegans* (An and Blackwell, 2003). The FOXO transcription factor DAF-16 is involved in metabolism, stress resistance, and modulation of longevity (Zečić and Braeckman, 2020). Additionally, plant products have been investigated for their effects on stress tolerance and longevity. For example, leaf extracts from *Anacardium occidentale*, impact oxidative resistance and extend lifespan. The study showed that the extracts function through the DAF-16/FoxO and SKN-1/Nrf-2 signaling pathways. Moreover, the study investigated the stimulation of stress response genes, including SOD-3 and GST-4. (Duangjan et al., 2019). Wang *et al.* demonstrated that *C. elegans* supplemented with orange extracts promotes longevity, thermal stress resistance, and enhances health span by upregulating levels of oxidative stress-related genes including daf-16, sod-3, gst-4, sek-1, and skn-1. (J. Wang et al., 2020). However, the effects of GAL extracts on *C. elegans* have not yet been explored.

In the context of the traditional uses of *G. atroviridis* leaf, particularly regarding its potential impact on weight management among obese or overweight individuals, a single sachet of dried *G. atroviridis* leaves was brewed in 200 mL of boiling water at 100°C and allowed to cool to an optimal drinking temperature before consumption (Lumbantobing et al., 2017a; Lumbantobing et al., 2017b; Shahid et al., 2022). More comprehensive studies, including clinical trials, have indicated that *G. atroviridis* leaf tea reduces body mass index and waist circumference, along with a significant decline in fasting blood glucose and triglyceride levels in adults with obesity (Lumbantobing et al., 2017a; Lumbantobing et al., 2017b). Abdominal adiposity, implicated in the aging process, is associated with elevated levels of pro-inflammatory cytokines, which can disrupt insulin action. Age-related alterations in body fat distribution and metabolism constitute significant factors in accelerating the aging process and precipitating age-related diseases (Jura et al., 2016). Obesity is further characterized by chronic low-grade inflammation concomitant with escalating oxidative stress. Excessive oxidative stress leads to structural damage within cells coupled with inadequate production of antioxidant mechanisms (Marseglia et al., 2014). Thus, elucidating the molecular mechanisms of antioxidants involved in mitigating oxidative stress and aging is imperative for promoting healthy aging and counteracting stress. Consequently, this study employed *C. elegans* as an *in vivo* model to investigate the biological effects of GAL extracts on longevity, anti-heat stress, and antioxidant properties. The novel aspects of this study included the evaluation of GAL extracts and their effects on oxidative stress, heat stress resistance, and longevity in *C. elegans*. This investigation stands out as it suggests that the beneficial effects of GAL extracts could be through the stimulation of oxidant enzymes mediated by DAF-16/FOXO and SKN1/NRF-2 pathways and by enhancing thermal defense. The study systematically reveals how these pathways play a role in countering heat stress and oxidative stress, which is linked to the aging process. The potential for GAL extract in the development of anti-aging nutritional supplements represents an exciting area for future research with practical implications in ethnopharmacology and related fields.

## 2 Materials and methods

### 2.1 Preparation of *Garcinia atroviridis* leaves

*G. atroviridis* leaves were obtained from Thasala, Nakhon Si Thammarat, Thailand. A voucher number (016443) was deposited at the herbarium of Kasin Savutabhandhu, Department of Botany, Faculty of Science, Chulalongkorn University, Thailand. Fresh leaves were thoroughly cleaned with water and then sun-dried for 3 days. The dried leaves were ground into a powder. A cellulose cartridge was filled with 10 g of the dried leaf powder and placed in the Soxhlet extraction apparatus (Chatatikun et al., 2019). Sequentially, the powder was extracted with 400 mL of petroleum ether, dichloromethane, and absolute ethanol, each for a duration of 24 h. The petroleum and dichloromethane extracts were discarded, while the ethanol extract was concentrated using a vacuum evaporator. Before use, the ethanol extract was dissolved in dimethyl sulfoxide to achieve a stock concentration of 100 mg/mL.

### 2.2 Determination of phenolic contents

The total phenolic content (TPC) of GAL was determined using the Folin-Ciocalteu method (Kooltheat et al., 2021). In brief, 50 µL of 10% Folin Ciocalteu reagent was added to 50 µL of the ethanol extract in a 96-well plate. The mixture was thoroughly combined, followed by the addition of 50 µL of 1 N sodium carbonate. After thorough mixing, the plate was incubated at room temperature for 1 h. The absorbance at 750 nm was measured against the blank using a microplate reader (BioTek Instruments Inc., United States of America). TPC results were expressed as milligrams of gallic acid equivalents per Gram of dry weight (mg GAE/g DW).

### 2.3 Determination of flavonoid contents

The total phenolic content (TPC) of GAL was determined using the Folin-Ciocalteu method (Kooltheat et al., 2021). In brief, 50 µL of 10% Folin Ciocalteu reagent was added to 50 µL of the ethanol extract in a 96-well plate. The mixture was thoroughly combined, followed by the addition of 50 µL of 1 N sodium carbonate. After thorough mixing, the plate was incubated at room temperature for 1 h. The absorbance at 750 nm was measured against the blank using a microplate reader (BioTek Instruments Inc., United States of America). TPC results were expressed as milligrams of gallic acid equivalents per Gram of dry weight (mg GAE/g DW).

### 2.4 Determination of 2,2-diphenyl-1-picrylhydrazyl (DPPH) radical scavenging activity

The radical scavenging activity of GAL was assessed using the DPPH assay (Chatatikun and Kwanhian, 2020). In this assay, 10 µL of the extract solution in methanol was mixed with 90 µL of 0.1 mM DPPH^•^ solution. The resulting mixture was incubated in a dark area for 30 min. Following incubation, the absorbance was measured at 517 nm against an equal volume of DPPH^•^ solution and methanol, serving as the blank. The percentage of DPPH^•^ scavenging activity for each sample was calculated using the equation:

$${\%\;DPPH}^{•\;}scavenging\;activity={\left[(A\right.}_{0}{-A}_{1}{)/A}_{0}]\times 100,$$

where A_0_ represents the absorbance of the control and A_1_ represents the absorbance of the extract. The extract concentration required to scavenge 50% of DPPH^•^ radicals was expressed as SC_50_ in µg/mL.

### 2.5 Determination of 2,2′-azino-bis-(3-ethylbenzothiazoline-6-sulphonic acid) (ABTS) radical scavenging activity

The ABTS scavenging activity was determined using a previously described method (Charoenphun and Klangbud, 2022). To prepare the ABTS radical cation (ABTS^•+^), a 7 mM ABTS stock solution was reacted with 2.45 mM potassium persulfate in a 2:3 ratio. The mixture was then left to stand in a dark area for 12–16 h at room temperature before use. The ABTS^•+^ solution was subsequently diluted with methanol to achieve an absorbance of 0.70 ± 0.02 at 734 nm. For the assay, 10 µL of the ethanol extract at various concentrations was mixed with 90 µL of the ABTS^•+^ solution. After incubation for 45 min, the absorbance was measured using a microplate reader. The ABTS^•+^ scavenging activity was calculated using the following equation:

$${\%\;ABTS}^{•+}\;scavenging\;activity={\left[(A\right.}_{0}{-A}_{1}{)/A}_{0}]\times 100,$$

where A_0_ represents the absorbance of the control and A1 represents the absorbance of the extract. The results were expressed as the half-maximal scavenging concentration (SC_50_) in µg/mL.

### 2.6 Liquid chromatography-quadrupole time-of-flight mass spectrometry (LC-QTOF MS) analysis

The metabolite profiles of GAL were determined using an LC-QTOF-MS instrument (Agilent 1290 Infinity LC instrument coupled to an Agilent 6540 series QTOF-MS equipped with an ESI source and a diode-array detector (DAD)) in positive ion mode. Chromatographic separation was conducted on an Agilent Poroshell 120 EC-C18 column (150 mm length x 4.6 mm inner diameter, particle size 2.7 μm). The gradient elution was performed using 0.1% formic acid water (mobile phase A) and acetonitrile (mobile phase B) at a flow rate of 200 μL/min. The injection volume was 1.0 μL, and the column temperature was maintained at 35°C. Data acquisition was controlled using the Mass Hunter Workstation Software Qualitative Analysis (version B.08.00, Agilent Technologies, California, United States of America). The phytochemical compounds present in the extract samples were identified by comparing retention times, mass data, and fragmentation patterns with a compound database in the library search of the Agilent MassHunter Personal Compound Database and Library (Agilent Technologies). Peaks with similarity scores of 80% compared to the database were selected to confirm peak identification (Sun et al., 2015; Zhu et al., 2022).

### 2.7 *Caenorhabditis elegans* strains, maintenance, and treatment

Wild type *C. elegans* (N2), CF1553 (muls84[pAD76(sod-3::GFP)]), (muls84[pAD76(sod-3::GFP)]), CL2166 (dvls19[pAF15(gst-4::GFP::NLS)]), were initially obtained from *Caenorhabditis* Genetics Center (CGC), United States of America. Worms were cultured on Nematode Growth media (NGM) and plates seeded with *Escherichia E.) coli* strain OP50 as a food source at 20°C (An and Blackwell, 2003). The worms were synchronized by the sodium hypochlorite method (Stiernagle, 2006). The L4 synchronized worms were transferred to a medium containing different concentrations of GAL extract at 25, 50, and 100 μg/mL, respectively. Worms treated with 0.01% DMSO were used as a vehicle control.

#### 2.7.1 Heat stress survival assay

Aged-synchronized L1 worms were treated with or without GAL extract for 48 h at 20°C and then transferred to 35°C. The survival rate was recorded hourly following the temperature change until no worms remained alive. The percentage survival rate was then calculated. Thirty worms were included in each group, and the experiments were conducted with a minimum of three repetitions.

#### 2.7.2 Oxidative stress resistance assay

Aged-synchronized worms treated with or without GAL extract for 48 h at 20°C were transferred to an NGM medium containing 0.003% H_2_O_2_ (Jin et al., 2020) and then the survival rate was observed and recorded every day. The percentage survival rates were then subsequently calculated. Thirty worms/groups were employed in the study and experiments were repeated at least three times.

### 2.8 Determination of reactive oxygen species (ROS)

The cellular ROS was quantified by the dichlorofluorescein assay (H. Wang and Joseph, 1999). The aged-synchronized L1 larvae of N2 worms were treated with GAL extracts or DMSO in S-medium at 20°C for 48 h. Cellular ROS levels were measured under oxidative stress conditions (exposed to 0.003% hydrogen peroxide for 12 h) and under heat stress conditions (exposed to 35°C for 2 h). Then, 50 μM H_2_DCF-DA was added and incubated for 1 h away from light at 20°C. The worms were then mounted on a glass slide and paralyzed by 10 mM sodium azide (NaN_3_), and 30 worms were photographed using a Carl Zeiss Axio Vert.A1 inverted microscope, with excitation at 488 nm and emission at 500–530 nm. The relative fluorescence intensities of the entire body were measured using ImageJ software.

### 2.9 Lifespan assay

Aged-synchronized worms were prepared, *and* approximately 150 worms were separately grown on 6 NGM agar plates (25 worms per plate) per treatment. Subsequently, the number of live worms was transferred to fresh plates and recorded daily until all worms had deceased. Lifespan analysis was conducted using log-rank analysis through OASIS, an Online Application for the Survival Analysis of Lifespan Assays Performed in Aging Research (Yang et al., 2011).

### 2.10 Expression of oxidative stress-related reporters

Aged-synchronized CF1553, CL2166 (dvls19[pAF15(gst-4::GFP::NLS)]). strains, inducible green fluorescent protein (GFP) reporters for *sod-3 and gst-4* were monitored after 48 h of incubation at 20°C on treatment plates. Worms were anesthetized by 10 mM sodium azide (NaN_3_) and were picked onto a glass slide pre-equipped with a thin layer of 2% agarose gel. Fluorescence imaging was performed with an *Axio Vert.A1* inverted microscope by Carl Zeiss (Germany), with excitation at 488 nm and emission at 500–530 nm. Fluorescence intensities were determined using ImageJ software. Three independent experiments were performed per assay and reporter strain.

### 2.11 Quantitative RT-PCR

The total RNA of worms was extracted for each strain by GENEzol™ reagent (Geneaid, Taiwan). The concentration of the collected RNA was determined using nanodrop (NanoDrop™ 2000/2000c Spectrophotometers, Thermo Scientific™, United States of America). The first-strand cDNA was synthesized by iScript™ Reverse Transcription Supermix (Bio-Rad, United States of America). After cDNA synthesis, quantitative RT-PCR (qPCR) was investigated using 5x H.O.T. FIREPol^®^ EvaGreen^®^ qPCR Mix PCR (ROX) (Solis BioDyne, Estonia). Relative gene expression was analyzed using the 2^−ΔΔCt^ method with *act-1* as a control gene. The qPCR primers used are shown in Table 3. For each tested gene, three replicates were tested per concentration.

### 2.12 Statistical analyses

For statistical analysis, at least three replicates were conducted. For survival curves, log-rank (Mantel-Cox) tests were used for analysis. Data were assessed using Student’s t-test for two groups and Tukey’s multiple comparison tests, one-way ANOVA for three or more groups where necessary. *p* values less than 0.05 were considered statistically significant. Data were expressed as the mean ± SD.

## 3 Results

### 3.1 Total phenolic and flavonoid contents

The extraction yield of the ethanol extract of GAL was 18.14% (w/w). The total phenolic content (TPC) of the ethanol extract of GAL was quantified using a calibration curve (y = 0.2017x–0.0519, *R* ^2^ = 0.992) of gallic acid (0–100 μg/mL) and expressed as gallic acid equivalents (GAE) per Gram dry extract weight. TPC in the ethanol extract was measured to be 7.69 ± 0.69 mg GAE/g dry weight extract.

The total flavonoid content (TFC) of the ethanol extract was determined using a calibration curve (y = 0.156x + 0.0169) of quercetin (0–100 μg/mL) and expressed as quercetin equivalents (QE) per Gram dry extract weight. The TFC in ethanol extract was found to be 8.83 ± 0.51 mg QE/g dry weight extract.

### 3.2 DPPH radical scavenging activity

**FIGURE 1:**
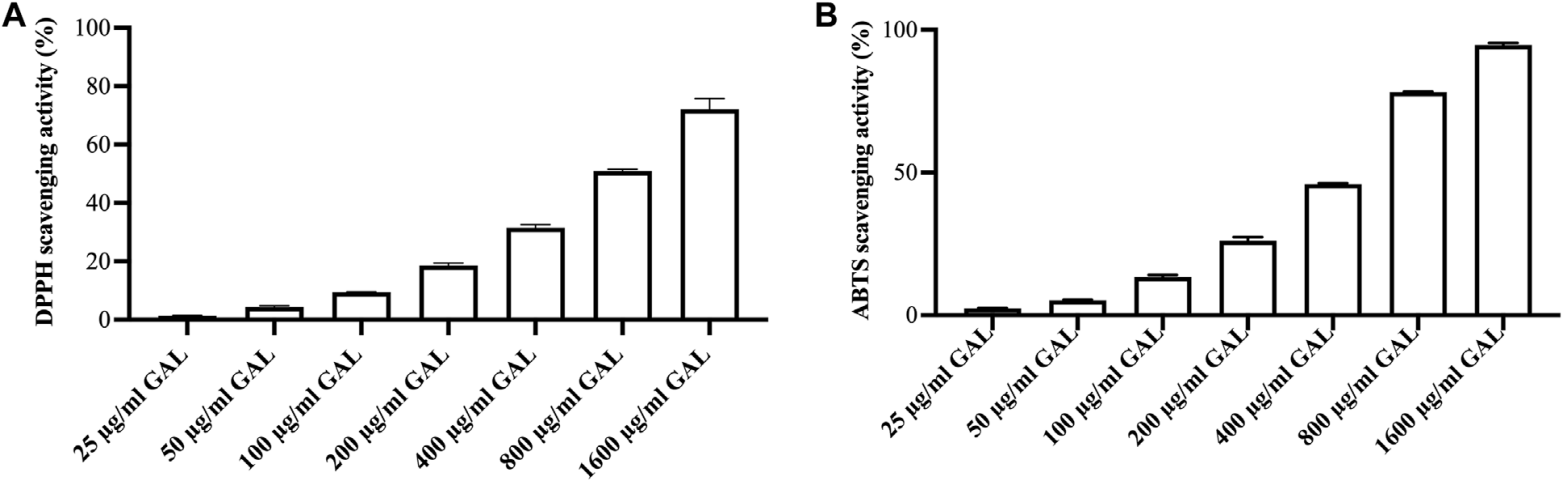
The radical scavenging activity. **(A)** DPPH radical scavenging activity of ethanol extract of GAL. Each column represents the mean ± SD, n = 3. **(B)** ABTS cation radical scavenging activity of ethanol extract of GAL. Data are expressed as the mean ± SD, n = 3.

DPPH assay was conducted to measure the relative antioxidant capability of the GAL extract in scavenging the DPPH radical. The results of the free radical scavenging activity of the ethanol extract of GAL are presented in Figure 1A. The GAL extract exhibited a concentration-dependent ability to scavenge the DPPH^•^ free radical up to a given concentration. The SC_50_ value of the ethanol extract was 970.37 ± 31.40 μg/mL, while the SC_50_ value of ascorbic acid was 2.20 ± 0.03 μg/mL. The results revealed that the ethanol extract of GAL possesses free radical properties.

### 3.3 ABTS cation radical scavenging activity

A concentration-response relationship was observed in the ABTS cation radical scavenging assay, where the scavenging activity of the GAL extract increased with higher concentrations Figure 1B. The intensity of the color is directly related to the presence of antioxidants in the extract. The SC_50_ values of the ethanol extract and ascorbic acid, representing the concentration at which 50% ABTS cation radical scavenging activity was 533.36 ± 7.65 μg/mL and 4.27 ± 0.17 μg/mL, respectively. These results indicate that the ethanol extract of GAL possesses the ability to interact with ABTS cation radicals.

### 3.4 LC-QTOF-MS analysis of *Garcinia atroviridis*

**FIGURE 2:**
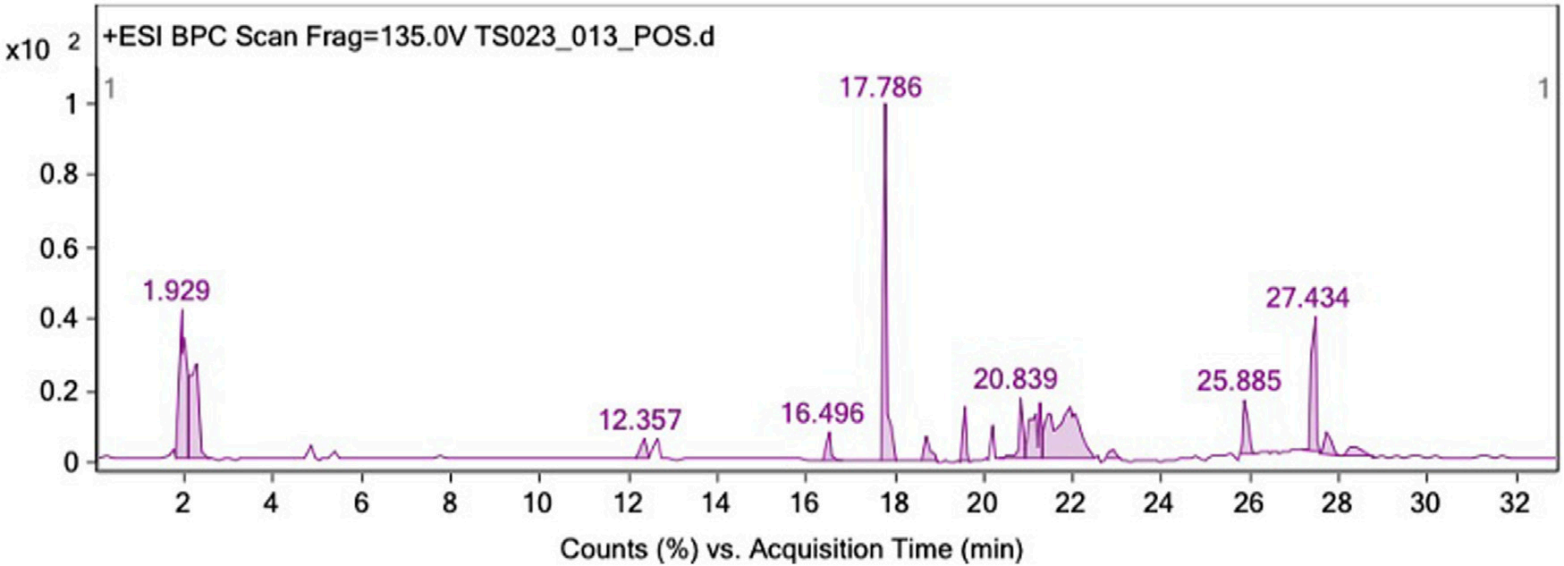
LC-QTOF-MS identification of GAL extract in the positive ion mode.

LC-QTOF-MS in positive mode was employed to conduct a qualitative analysis of the compounds present in GAL. The chromatographic peaks corresponding to the compounds in the crude extract were tentatively identified by comparing the MS data with the Agilent MassHunter Workstation software and the Personal Compound Database and Library (PCDL), based on the search of molecular ion peaks in the positive mode [M + H]+. A total of 18 compounds were identified from the ethanolic extract of GAL using LC-QTOF-MS. These compounds included 12 xanthones (garcinexanthone F, morellin dimethyl acetal, malcurin, nigrolineaxanthone A, nigrolineaxanthone P, bannaxathone A, garcimangosone B, scortechinones Q, scortechinone V, moreollic acid, gambogin), one flavone (dulcinoside), two phloroglucinols (vismiaphenone C, garcinielliptone R) and two polyprenylated cyclic polyketides (guttiferone K and oxy-guttiferone K2) derived from GAL. The list of analyzed compounds using LC-QTOF-MS is presented in Table 1, supported by Figure 2.

**TABLE 1:** Compounds identified in the LC-QTOF-MS of ethanolic *Garcinia atroviridis* extract.

No.	RT (min)	Molecular weight	m/z	Molecular formula	Mass error (ppm)	Tentative identification	Classification
1	2.391	356.053	357.060	C_18_H_12_O_8_	0.51	Garcinexanthone F	Xanthones
2	5.255	230.224	253.213	C_14_H_30_O_2_	−1.37	Morellin dimethyl acetal	Xanthones
3	16.496	262.047	285.036	C_13_H_10_O_6_	−2.68	Maclurin	Xanthones
4	16.714	578.163	579.170	C_27_H_30_O_14_	−0.34	Dulcinoside	Flavones
5	18.148	344.125	362.159	C_19_H_20_O_6_	−1.28	Nigrolineaxanthone A	Xanthones
6	19.318	414.168	437.157	C_23_H_26_O_7_	1.8	Bannaxanthone A	Xanthones
7	19.561	408.155	409.162	C_24_H_24_O_6_	−4.79	Garcimangosone B	Xanthones
8	19.561	444.216	462.249	C_25_H_32_O_7_	4.42	Nigrolineaxanthone P	Xanthones
9	21.923	578.285	579.293	C_34_H_42_O_8_	−3.59	Scortechinones Q	Xanthones
10	22.720	380.198	398.232	C_24_H_28_O_4_	−1.5	Vismiaphenone C	Phloroglucinols
11	22.827	606.246	607.252	C_34_H_38_O_18_	−0.52	Scortechinone V	Xanthones
12	23.982	608.263	609.270	C_34_H_40_O_10_	1.41	Scortechinones C	Xanthones
13	24.228	602.362	620.396	C_38_H_50_O_6_	2.63	Guttiferone K	Polyprenylated cyclic polyketides
14	24.228	592.270	615.257	C_34_H_40_O_9_	4.93	Moreollic acid	Xanthones
15	24.305	598.327	616.360	C_38_H_46_O_6_	−3.66	Gambogin	Xanthones
16	24.854	600.345	618.379	C_38_H_48_O_6_	1.16	Oxy-guttiferone K2	Polyprenylated cyclic polyketides
17	26.939	518.324	541.313	C_30_H_46_O_7_	0.81	Garcinielliptone R	Phloroglucinols

### 3.5 GAL extracts increased lifespan in *C. elegans*

**FIGURE 3:**
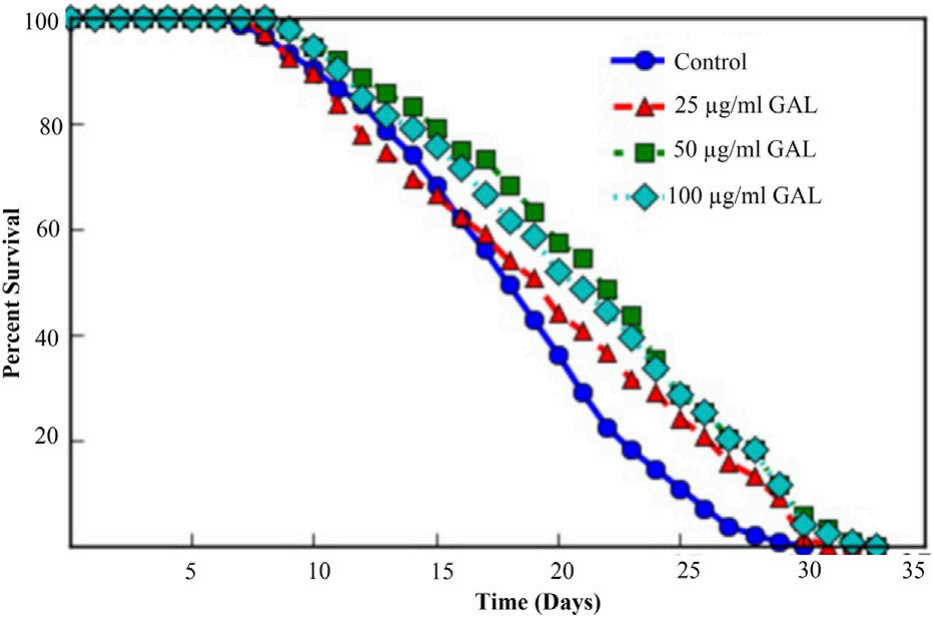
Effect of GAL extracts on the lifespan of N2 wild type *C. elegans.* Synchronized worms were grown on NGM-OP50 plates with DMSO or 25, 50, or 100 μg/mL GAL extracts at 20 °C (n = 150 worms/group). Statistical differences compared to control (DMSO) by using log-rank test.

In this study, the *C. elegans* model was utilized to investigate the effect of GAL extract on lifespan*. C. elegans* is an excellent model for studying aging, drug toxicity, and environmental stress and the result will be reflected the longevity. The initial selection of doses for the experiments was carried out through the screening of previous studies on plant extracts (Rangsinth et al., 2019). Following this, the first phenotype test was performed to assess the impact of GAL extracts on the longevity of *C. elegans*, utilizing concentrations of 25, 50, and 100 μg/mL. For the first time, we demonstrated that GAL extracts extended the mean lifespan. N2 worms were treated with GAL extracts at concentrations of 25, 50, and 100 μg/mL, respectively. As depicted in Figure 3, the survival rate of GAL-treated worms was significantly monitored daily until all worms died. The results indicated that GAL extracts notably increased the mean lifespan of *C. elegans* by 6.02%, 15.26%, and 12.75% at concentrations of 25, 50, and 100 μg/mL, respectively, compared to the control groups treated with DMSO (Figure 3; Table 2). The extension of lifespan was surprisingly found to not be dependent on the dosage. Consequently, we carried out further experiments using these specific concentrations.

**TABLE 2:** Effect of GAL extracts on the lifespan of N2 wild type *C. elegans*.

Groups	Number of worms	Mean lifespan (Days)	Age in days at 100% mortality	*p*-value compare to N2
Control	150	18.27 ± 0.46	30	—
25 μg/mL GAL	150	19.44 ± 0.61	31	<0.01
50 μg/mL GAL	150	21.56 ± 0.57	33	<0.01
100 μg/mL GAL	150	20.94 ± 0.59	33	<0.01

*p < 0.01*, compared with control (N2 treated with DMSO).

### 3.6 GAL extracts enhanced stress tolerance in *C. elegans*

**FIGURE 4:**
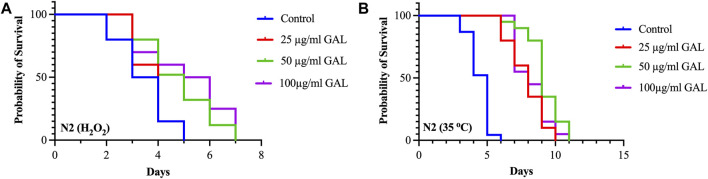
Effect of GAL extracts treatment on stress tolerance of wild type *C. elegans*. Synchronized worms were cultured on NGM-OP50 plates containing DMSO or 25, 50, and 100 μg/mL GAL extracts at 20°C until the L4-larvae stage. **(A)** Oxidative stress survival of worms exposed to 0.003% hydrogen peroxide at 20°C (n = 20 worms/group). **(B)** Heat stress survival of worms exposed to heat at 35°C (n = 20 worms/group).

To investigate the effects of GAL on the stress tolerance of *C. elegans*, the GAL extract treatments were tested for resistance to H_2_O_2_ oxidative stress and heat stress (35°C). We observed that the survival rate of the GAL treatment groups was increased compared to the control group. To provide further insights into these findings, we observed behavior of *C. elegans* and health status during the stress tolerance assays. These observations revealed that worms treated with GAL extract exhibited enhanced vitality and locomotion compared to the control group. Additionally, assessments of worm behavior indicated a reduced incidence of stress-related behaviors, such as slow or no movement, in the GAL extract-treated groups under both H_2_O_2_ oxidative stress and heat stress conditions. Under oxidative stress conditions, *C. elegans* exposed to 0.003% hydrogen peroxide oxidative stress observed stress tolerance by increasing the average lifespan in GAL extract-treated compared to the control group (Figure 4A; Table 3). Furthermore, we observed a delayed onset of oxidative stress-induced appearance, such as diminished body size, in GAL extract-treated worms compared to controls. Moreover, in the heat stress survival assay, worms were cultured at 35°C and counted survival worms every day until all worms died. The average lifespan of *C. elegans* exposed to heat resulted in a significant increase in the survival of GAL extract-treated compared to the control, as shown in (Figure 4B; Table 4). Observations during the heat stress assay highlighted a higher proportion of active and motile worms in the GAL extract-treated groups, indicating improved heat stress resistance and vitality. These combined results indicate that GAL extract potentially could protect the worms under H_2_O_2_ and 35°C stress conditions, suggesting a promising avenue for further investigation into its mechanisms of action.

**TABLE 3:** The primer sequence of the genes.

Genes	Forward primer 5'->3′	Reverse primer 5'->3′
*act-1*	ACG​ACG​AGT​CCG​GCC​CAT​CC	GAA​AGC​TGG​TGG​TGA​CGA​TGG​TT
*sod-3*	ATC​TAC​TGC​TCG​CAC​TGC​TT	TTT​CAT​GGC​TGA​TTA​CAG​GTT
*gst-4*	GCC​CGT​GAT​GAT​TTC​TTG​GC	GCC​CAA​GTC​AAT​GAG​TCT​CCA
*hsp-16.2*	TGT​TGG​TGC​AGT​GCT​TCG​AAT​C	TTC​TCT​TCG​ACG​ATT​GCC​TGT​TG

**TABLE 4:** Effect of GAL extracts on stress tolerance in *C. elegans*.

Tests	Groups	Median survival (Day)	*p*-value compared to N2
Oxidative Stress	Control	3.5	—
	25 μg/mL GAL	4.0	0.4643
	50 μg/mL GAL	5.0	0.0015
	100 μg/mL GAL	5.5	0.004
Heat Stress	Control	5.0	-
	25 μg/mL GAL	8.0	<0.0001
	50 μg/mL GAL	9.0	<0.0001
	100 μg/mL GAL	8.0	<0.0001

### 3.7 GAL extracts decreased intracellular ROS in *C. eleg*ans

**FIGURE 5:**
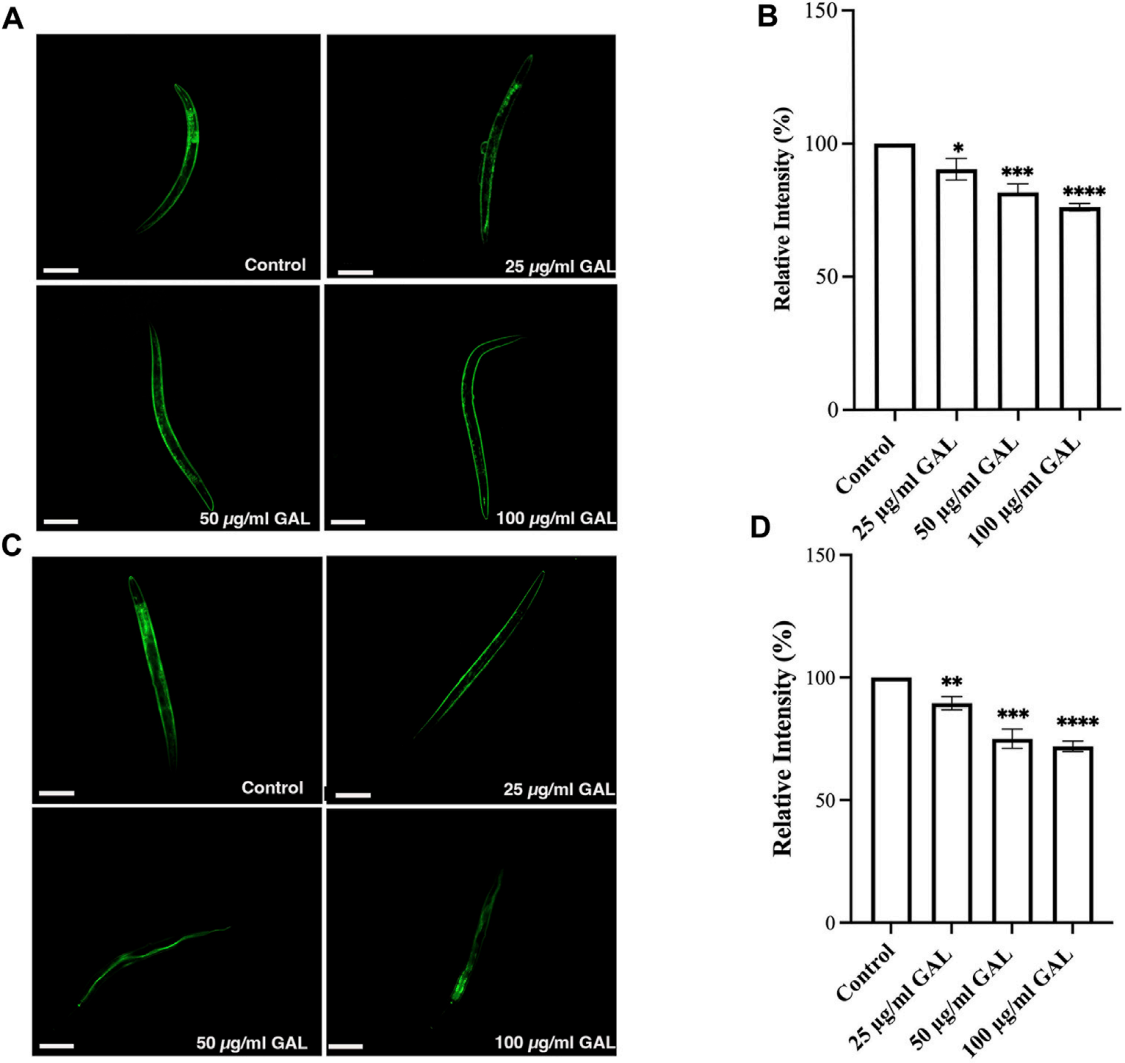
GAL Extracts decreased intracellular ROS in *C. elegans*. The size of the scale bar measurement is 100 μm. **(A)** Cellular ROS levels under Oxidative stress conditions (exposed to 0.003% hydrogen peroxide) were compared between untreated control and worms treated with 25, 50, and 100 μg/mL GAL extracts (n = 30 worms/group). **(B)** Relative percent expression of oxidative stress conditions was shown compared to 100% for untreated control. **(C)** Cellular ROS levels under heat stress conditions (exposed to heat at 35°C) were compared between untreated control and worms treated with 25, 50, and 100 μg/mL GAL extracts (n = 30 worms/group). **(D)** Relative percent expression of heat stress conditions was shown compared to 100% for untreated control. Statistical differences compared to the control (DMSO) were considered significant. *p < 0.05, ***p < 0.001, ****p < 0.0001. The error bars represent the standard deviation (SD).

*C. elegans* was employed as a model organism to study the potential antioxidant properties of the ethanol extract of GAL *in vivo*. In our investigation, GAL extract demonstrated an enhancement in *C. elegans* survival rates under both heat stress and H_2_O_2_-induced oxidative damage conditions. To provide a comprehensive understanding of these findings, the data was collected through intracellular ROS level measurements using H_2_DCF-DA, a widely utilized fluorescence probe for detecting ROS production (Tarpey and Fridovich, 2001). The fluorescence intensity resulting from ROS-induced oxidation causes the formation of 2′,7′-Dichlorodihydrofluorescein, which is directly proportional to the ROS levels. Under oxidative stress conditions, worms were subjected to treatment with GAL extract and 0.003% or H_2_O_2_. Remarkably, the results revealed a significant reduction in fluorescence intensity in worms pre-treated with GAL extract compared to the untreated control. Moreover, from the intensity analysis indicated that pre-treatment with GAL extract led to a notable decrease in intracellular ROS levels under heat stress at 35°C (Figure 5), providing further evidence of its antioxidant properties.

### 3.8 GAL extracts increased the expression of SOD-3 and GST-4

**FIGURE 6:**
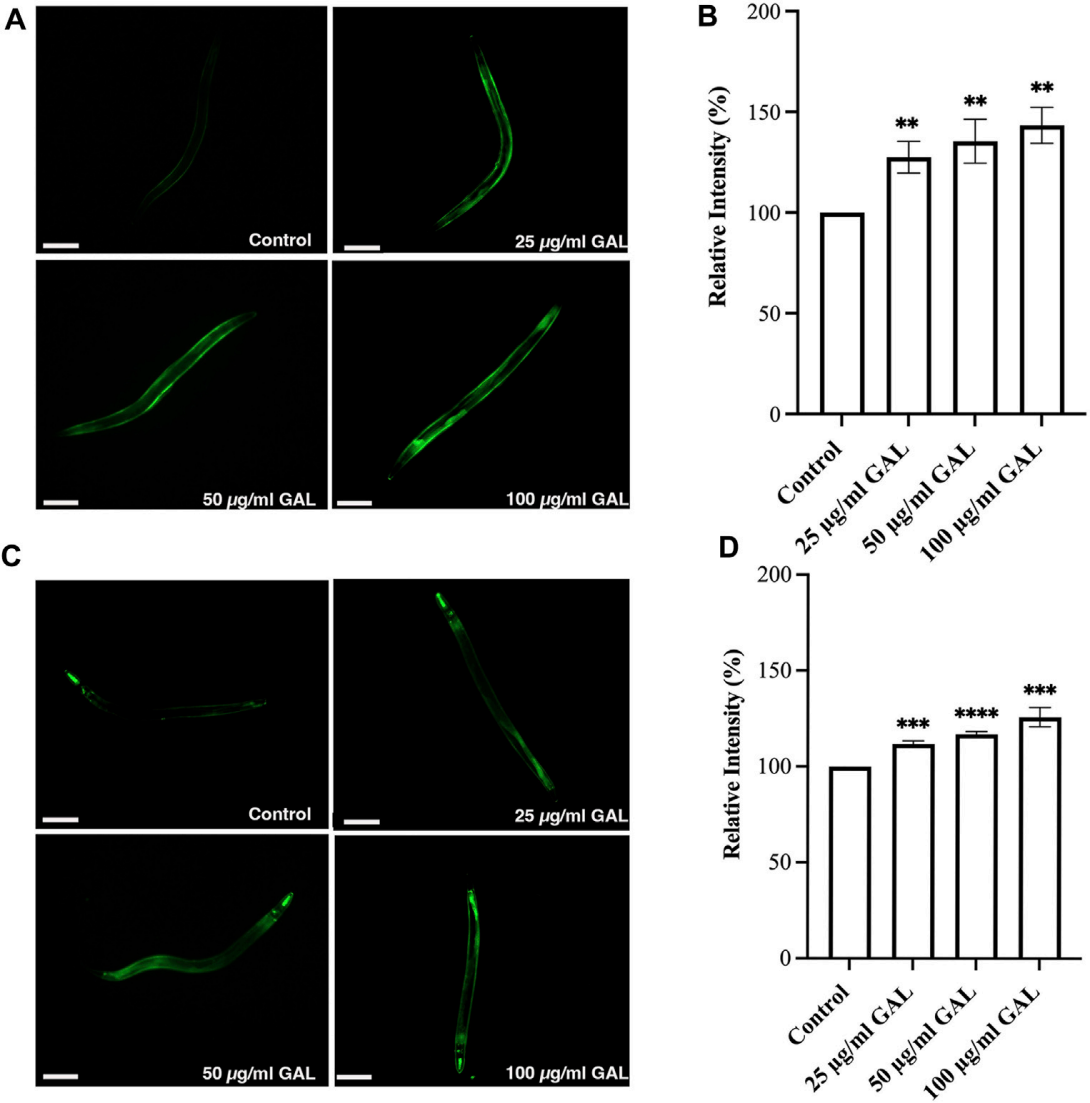
Effect of GAL extract on GST-4 and SOD-3 expression in *C. elegans*. The size of the scale bar measurement is 100 μm. **(A)** GFP expression induced by *gst-4* promoter was observed in a fluorescence microscope. **(B)** The relative percent expression of *gst-4* was shown compared to 100% for untreated control. **(C)** GFP expression induced by *sod-3* promoter was observed in a fluorescence microscope. **(D)** The relative percent expression of *sod-3* was compared to 100% for untreated control (n = 30 worms/group). Statistical differences compared to the control (DMSO) were considered significant. *p < 0.05, **p < 0.01, ***p < 0.001, ****p < 0.0001. The error bars represent the standard deviation (SD).

Previous research has indicated that GST-4 and SOD-3 expression levels are positively associated with lifespan in *C. elegans* and can serve as a biomarker for longevity. To further investigate the underlying mechanisms, we examined the expression of the gst-4 gene using the transgenic worm strains CL2166 (dvIs19[pAF15(gst-4::GFP::NLS)]). GST-4 is a member of the glutathione S-transferase family involved in oxidative stress responses. In addition to quantitative measurements, qualitative data was gathered through visual inspection of fluorescence intensity in transgenic worms. Supplementation with 25, 50, and 100 μg/mL GAL extracts significantly upregulated the expression of gst-4, as evidenced by increased GFP fluorescence intensity of CL2166 transgenic worms (Figures 6A, B). This assessment provided visual confirmation of the enhanced expression of gst-4 in response to GAL extract treatment. Furthermore, we evaluated the expression of the sod-3 gene using the transgenic worm strains CF1553 (muIs84[pAD76(sod-3::GFP)]). Superoxide dismutase-3 (SOD-3) is an enzyme crucial for maintaining oxidation-reduction balance. Similarly, qualitative analysis of fluorescence intensity revealed higher expression levels of SOD-3:GFP in CF1553 transgenic worms treated with 25, 50, and 100 μg/mL GAL extract, respectively (Figures 6C, D). The observation confirmed the increase in sod-3 expression with GAL extract, showing a dose-dependent effect. Consequently, this evidence visually supports the enhanced expression of gst-4 and sod-3 due to GAL extract treatment, providing insight into its antioxidant mechanisms and potential impact on lifespan regulation in *C. elegans*.

### 3.9 GAL extracts increased oxidative stress-related genes in *C. elegans*

**FIGURE 7:**
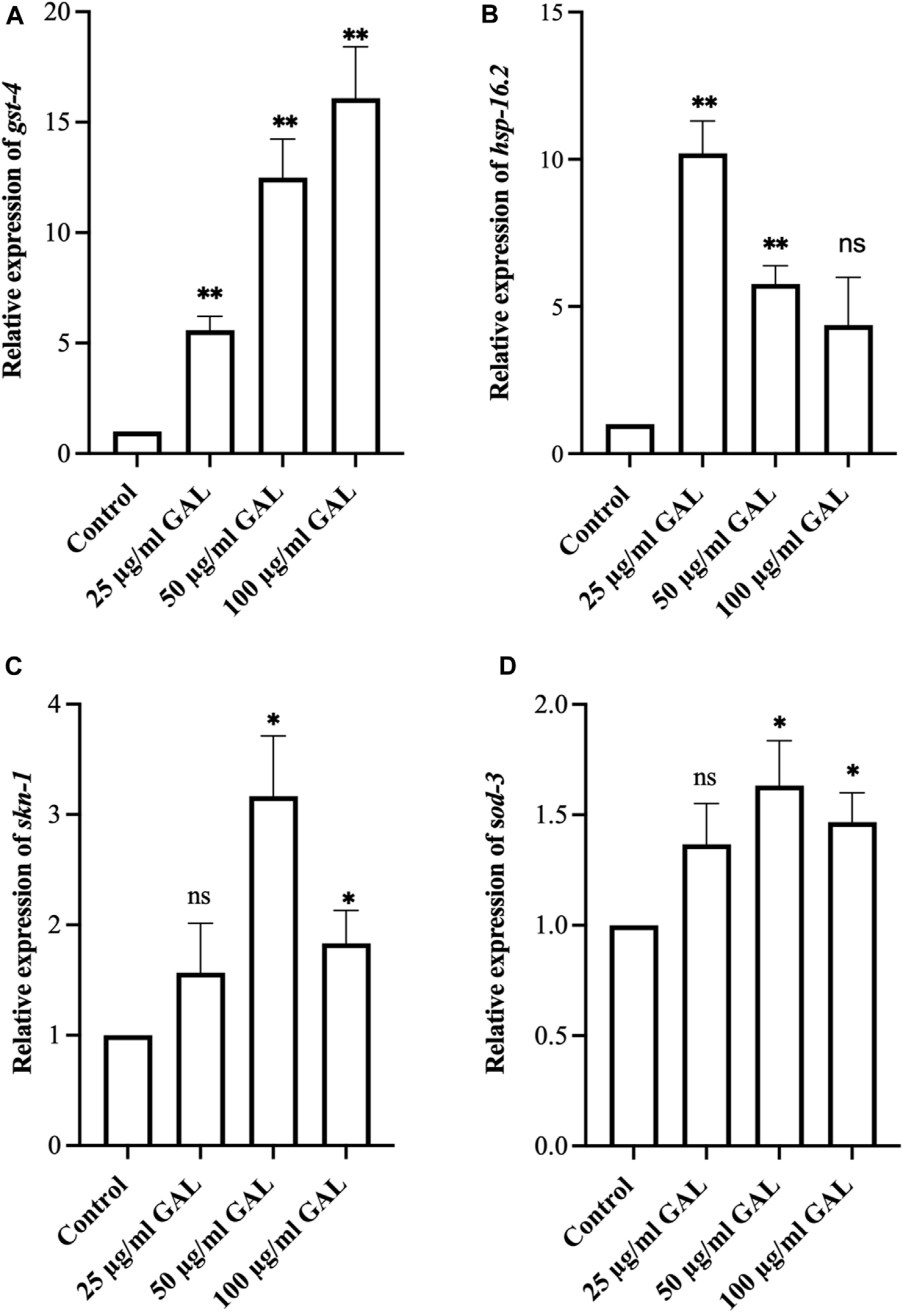
Effect of GAL extract on oxidative stress-related genes in *C. elegans*. **(A–D)** The relative mRNA expression of *gst-4*, *hsp16.2, skn-1, and sod-3,* respectively. Statistical differences compared to control (DMSO) were considered significant. *p < 0.05, **p < 0.01, and ns as non-significant. The error bars represent the standard deviation (SD).

To investigate the antioxidant mechanisms of GAL extracts, we employed both molecular analysis and visual observations to evaluate mRNA expression levels of oxidative stress-related genes in *C. elegans*. Upon observing the increased expression of transgenic sod-3 and gst-4 post-GAL extract treatments, our data suggested a potential regulatory mechanism mediated via the SKN-1/NRF2 pathway. As depicted in Figure 7, analysis revealed a significant rise in gst-4, skn-1, hsp16.2, and sod-3 expression following GAL extract treatment, providing visual evidence of the regulatory effects exerted by GAL extracts. This observation underscores the potential of GAL extract to protect *C. elegans* from severe oxidative stress and reduce intracellular ROS levels through activation of the SKN-1/NRF2 pathway. The transcription factors SKN-1/NRF2 and DAF-16/FOXO play vital roles in responding to oxidative stress, with GST-4 and SOD-3 among the antioxidant enzymes they regulate. SKN-1, analogous to the mammalian NRF protein, is essential for inducing the expression of phase-II detoxification enzymes and antioxidant proteins such as SOD, GST, and glutathione peroxidase. In our study, we anticipated observing an enhancement in the expression of these related genes. Through analysis, we indeed identified a significant increase in mRNA levels of gst-4 and skn-1 expression compared to act-1 as a control. (Figures 7A, C, D). Additionally, GAL extracts elevated the mRNA level of hsp 16.2, as illustrated in Figure 7B. This upregulation suggests a potential mechanism by which GAL extracts enhance heat stress survival and lifespan. HSP proteins, functioning as molecular chaperones and proteases, play a vital role in preventing oxidized or degraded proteins from aggregating prior to refolding or degradation (Hsu et al., 2003). The increased expression of hsp 16.2 induced by GAL extracts may contribute to maintaining protein homeostasis and cellular integrity under heat stress conditions, further supporting the beneficial effects of GAL extracts on *C. elegans* health span and stress resistance.

## 4 Discussion

Natural compounds have emerged as promising candidates for novel antiaging agents, with a growing body of research highlighting correlations between natural antioxidant compounds and their antiaging effects. Notably, this study marks the inaugural investigation into the antiaging potential and oxidative stress resistance properties of GAL observed in *C. elegans*. GAL extracts from fruits, roots, stems and barks have major compounds as organic acids and flavonoids. These compounds are present in the foods such as fruits, vegetables, spices, and medicinal plants (Shahid et al., 2022). In the previous study, the aqueous extract of GAL prepared at 100°C contained the highest phenolic content (20.21 ± 0.21 mg/dL) (Nursakinah et al., 2012). In other studies, the water and methanol extracts of GAL were 29.93 ± 0.43 and 18.69 ± 0.60 mgGAE/100g samples (Rabeta and Nur Faraniza, 2013). However, the ethanol extract of GAL has not yet been reported about total phenolic and flavonoid contents. Our present study revealed that the ethanol extract of GAL contains phenolic and flavonoid contents. The free radical activity of GAL is attributed to its phytochemicals. The ethanol extract of GAL exhibited free radical scavenging activity using DPPH and ABTS assays. The active compounds identified in the GAL extract through LC-QTOF-MS in our study include xanthones, flavone, phloroglucinols, and polyprenylated cyclic polyketides. This finding represents the first report on the phytochemical profiling in *G. atroviridis* fruit extracts belonging to the classes of organic acids, xanthones, terpenoid, sesquiterpenoid, and prenylated xanthone. Garcineflavonol A, garineflavanone A, garcinexanthone G were identified in the stem bark extract of *G. atroviridis* (Yamaguchi et al., 2000; Liu et al., 2015; Reang et al., 2021; Shahid et al., 2022).

Using *C. elegans* as a model organism is advantageous for a variety of phenotypic studies, especially in the context of aging and longevity research. We initially selected all doses for the experiments by screening plant extract research (Rangsinth et al., 2019). We then conducted the first phenotype test on the lifespan of *C. elegans* at concentrations of 25, 50, and 100 μg/mL of GAL extracts. Results showed that GAL extracts increased longevity by 6.02%, 15.26%, and 12.75% at doses of 25, 50, and 100 μg/mL, respectively. Unexpectedly, the lifespan extension was not dose-dependent, we then performed other experiments with these concentrations (Figure 3). Our data support the previous finding whereby the scavenging activity of the extract proves the presence of phenolic and flavonoid contents as free radical inhibitors (Nursakinah et al., 2012; Al-Mansoub, Asmawi, and Murugaiyah, 2014). The plant extracts are composed of phytochemical compounds and have antioxidant activity in several studies that enhance the lifespan of *C. elegans*. For example, Wang et al. showed that *C. elegans* supplemented with orange extracts promotes longevity. Leaf extract of *Caesalpinia mimosoides* was reported the antioxidant properties and an increased lifespan of *C. elegans* (Rangsinth et al., 2019). In *C. elegans*, the extended longevity was evidenced by anti-stress effects. For example, blueberry which has rich in bioactive phytochemicals increases lifespan and enhances stress resistance (Wang et al., 2018). Moreover, Song et al. showed an increase in lifespan treated with raspberry extract, which was accompanied by anti-stress effects in *C. elegans* (Song et al., 2020). In this study, we investigated whether the GAL extracts improved heat and oxidative stress in *C. elegans*. The effects of GAL extracts on the stress tolerance of *C. elegans*, the GAL extracts were tested for resistance to heat stress (35°C) and H_2_O_2_ oxidative stress. In the heat stress test, results revealed that GAL extract extended the lifespan of *C. elegans*. The results indicated that GAL extract potentially could protect the worms under 35°C and H_2_O_2_ stress conditions (Table 4). Moreover, to test whether heat stress tolerance is a beneficial effect of GAL extracts, the gene expression of hsp gene was determined in *C. elegans*. Previous reports revealed that high HSP (Heat shock protein) levels improve lifespan and indicate the capacity to resist heat stress (Rea et al., 2005; Zhang et al., 2009). Because it is closely correlated to heat tolerance and longevity, the HSP protein family was employed as a predictor of lifespan in *C. elegans* (Lin et al., 2021). By minimizing the accumulation of aggregated proteins in response to heat and other kinds of stress, HSP serves as molecular chaperones and proteases. This function may prevent oxidized or otherwise degraded proteins from aggregating prior to refolding or degradation (Hsu et al., 2003). In this work, GAL extracts raised the mRNA level of hsp 16.2 in *C. elegans* (Figure 7B), which may explain why it significantly increased heat stress survival and lifespan. Other studies have similarly linked the increase in lifespan and thermal stress resistance in *C. elegans* produced by various polyphenols to the ability to upregulate hsp and other genes involved with stress resistance. Additionally, we hypothesized that oxidative stress induced by H_2_O_2_ might increase the genes that are involved in oxidative stress in *C. elegans.*

*C. elegans* is substantiated by the use of various transgenic strains that have established roles in researching aging and stress response pathways. These strains possess genetic reporters that enable the observation of specific molecular pathways when the nematodes are exposed to the GAL extract being studied. We used gene reporters in *C. elegans*, including transgenic strains carrying GFP or other fluorescent proteins under the control of tissue-specific or inducible promoters. According to several studies, the anti-stress and longevity genes sod-3 and gst-4 were activated, which improved the anti-aging capacity of *C. elegans*. We then further investigated the mechanism underlying the antioxidant effect of GAL extracts, the expression of SOD-3 and GST-4 were initially evaluated by the GFP-reporter assays using GFP transgenic worms. Our present study showed that GAL extracts enhanced the GFP expression of SOD-3 and GST-4 (Figure 6). In concordance with the findings of the prior research, Luo et al. found that olive leaf extract exhibits antioxidant properties and the ability to increase the antioxidant activity of SOD and GSH-Px in *C. elegans* (Luo et al., 2019). Our GFP-reporter tests suggested that antioxidant enzymes may play a role in the protection of GAL extract treatments in *C. elegans*.

Furthermore, the transcription factors SKN-1/NRF2 and DAF-16/FOXO are two crucial players in the responses to oxidative stress. GST-4 and SOD-3 expression was examined among the antioxidant enzymes coded by SKN-1/NRF2 and DAF-16/FOXO. The transcription factor SKN-1 is the homolog of the mammalian NRF protein, which is crucial for stimulating the expression of phase-II detoxification enzymes and antioxidant proteins, such as SOD, GST, and glutathione peroxidase (GPO) (Luo et al., 2019). We expected to observe the enhancement of these related gene expressions. In addition, we then performed the qRT-PCR to investigate the gene expression. As a result, our study identified a significant increase in mRNA levels of gst-4 and skn-1 expression (Figure 7). Additionally, GAL extracts increased the mRNA level of sod-3, a downstream target of DAF-16, a FOXO transcription factor regulating the expression of antioxidant genes in *C. elegans* (Leonov et al., 2015). Taken together, GAL extracts may be related to the induction of antioxidant enzymes which may be mediated through DAF-16/FOXO and SKN-1/NRF2 in *C. elegans*. Collectively, these findings supported that GAL extracts with high antioxidant properties could protect the worms and prolong the average lifespan under stress conditions.

These findings not only advance our comprehension of the molecular mechanisms underlying stress resistance and aging but also pave the way for future investigations of specific active compounds of GAL and into potential therapeutic applications with implications for human health.

## 5 Conclusion

In conclusion, these findings suggest that GAL extracts exhibited stress-relieving properties, leading to stress resistance and longevity. The GAL extracts have chemical constituents according to qualitative analysis of LC-QTOF-MS. The protective effects of GAL extract against stress could be mediated through the regulation of DAF-16/FOXO and SKN-1/NRF2 pathways in *C. elegans*. Results highlight the potential uses of GAL extract as an antioxidant, anti-heat, and anti-aging treatment. However, further evidence from in-depth investigations utilizing mammalian models and active compounds of GAL extract is needed to confidently estimate the biotransformation pathways of GAL extract in mammals, including humans.

## Data Availability

The datasets presented in this study can be found in online repositories. The names of the repository/repositories and accession number(s) can be found in the article/Supplementary Material.
